# Analysis of Clinical Impact of CD33 rs12459419 Single-Nucleotide Polymorphism in AML Treated with Intensive Chemotherapy Without Gemtuzumab Ozogamicin

**DOI:** 10.3390/ijms27094050

**Published:** 2026-04-30

**Authors:** Sophie Helfenstein, Inna Shaforostova, Katja Seipel, Marie-Noelle Kronig, Myriam Legros, Ulrike Bacher, Thomas Pabst

**Affiliations:** 1 Department of Medical Oncology, Inselspital, Bern University Hospital, 3010 Bern, Switzerland; sophie.helfenstein@students.unibe.ch (S.H.); innaivanova.shaforostova@insel.ch (I.S.); katja.seipel@insel.ch (K.S.); marie-noelle.kronig@insel.ch (M.-N.K.); 2 Department for Biomedical Research, University of Bern, 3008 Bern, Switzerland; 3 Department of Hematology, Inselspital, Bern University Hospital, 3010 Bern, Switzerland; myriam.legros@insel.ch (M.L.); veraulrike.bacher@insel.ch (U.B.)

**Keywords:** acute myeloid leukemia (AML), CD33, rs12459419, single nucleotide polymorphism (SNP)

## Abstract

The CD33 rs12459419 (C>T; Ala14Val) single-nucleotide polymorphism (SNP) has been reported to modulate treatment response and survival in pediatric patients with acute myeloid leukemia (AML) receiving gemtuzumab ozogamicin (GO), an anti-CD33 antibody linked to the cytotoxic compound calicheamicin. However, it remains unclear whether this SNP also affects CD33 expression on leukemic blasts. Moreover, its prognostic significance in adult AML patients treated with standard chemotherapy without GO has not been investigated. In this study, we retrospectively genotyped 184 adult AML patients who received standard induction chemotherapy for the CD33 *rs12459419* SNP genotype and collected CD33 expression data. The observed genotype distribution was 46% (*n* = 85) CC, 43% (*n* = 79) CT, and 11% (*n* = 20) TT. CD33 expression was detected in significantly higher proportions of leukemic blasts in patients with the CC genotype than those with the TT genotype (***p* = 0.0009**). A similar trend was observed between the CT and TT genotypes (*p* = 0.06). No significant differences in clinical outcome were detected among the three genotype cohorts. Grouping CC and CT genotypes together based on their similar CD33 expression and comparing them to patients with the TT genotype also revealed no differences in overall survival (OS), event-free survival (EFS), or relapse-free survival (RFS). Using a proportion of 90% CD33-positive blasts to define high versus low expression groups also failed to identify a meaningful impact on OS, EFS, or RFS, either across genotypes or independent of genotype. In conclusion, our findings indicate that the CD33 *rs12459419* SNP does not affect outcomes or survival in adult AML patients receiving standard chemotherapy in the absence of GO. Furthermore, no association was seen between CD33 expression and clinical outcomes between the three genotypes. To our knowledge, this is the first study to investigate the prognostic impact of the CD33 *rs12459419* SNP per se on outcome and survival in adult AML patients treated with chemotherapy without GO. Validation in larger patient cohorts is required to conclusively rule out a prognostic role of the CD33 *rs12459419* SNP in AML.

## 1. Introduction

Acute myeloid leukemia (AML) remains a clinically challenging disease, with outcomes strongly affected by age, comorbidities, and disease biology. Although several new therapeutic agents have been approved in recent years, intensive chemotherapy remains a cornerstone of curative treatment for most adult patients. For medically fit individuals, standard induction therapy consists of cytarabine combined with an anthracycline (e.g., daunorubicin or idarubicin), the so-called “7+3” regimen. Depending on the patient’s molecular and cytogenetic profile, additional agents may be incorporated into induction treatment [1,2]. After complete remission, consolidation therapy typically involves further cycles of chemotherapy or hematopoietic stem cell transplantation (HSCT). Based on patient characteristics, AML risk stratification, and donor availability, either allogeneic (allo-HSCT) or autologous (auto-HSCT) transplantation is performed [2], with the latter being a safe alternative for patients with favorable-risk disease and in selected patients with intermediate-risk AML [3,4,5]. To further reduce relapse risk, maintenance therapy may be administered in selected patients [1].

Targeted therapies have also reshaped the AML treatment landscape. Among these, gemtuzumab ozogamicin (GO) is a humanized anti-CD33 monoclonal antibody conjugated to the cytotoxic agent calicheamicin, forming an antibody–drug conjugate (ADC). CD33, a sialic acid-binding immunoglobulin-like lectin (Siglec), is a myeloid differentiation antigen expressed on AML blasts and hematopoietic cells in the majority of AML cases, making it a suitable therapeutic target [6,7].

Clinical trials have demonstrated that adding GO to standard induction therapy improves overall survival (OS) and relapse-free survival (RFS) in adults with favorable- or intermediate-risk CD33-positive AML [8]. Further, adding GO to standard chemotherapy did not compromise stem cell mobilization success and therefore does not interfere with using auto-HSCT as a consolidation strategy [9].

CD33 expression shows heterogeneity across AML subgroups and is associated with specific molecular and cytogenetic subgroups, including *NPM1* mutations and *FLT3-ITD* mutations, whereas complex karyotype and CBF-AML are associated with lower CD33 expression [6,10,11]. High CD33 expression has been reported as an independent adverse prognostic factor in AML and is associated with significantly worse overall survival in some studies. However, other trials did not confirm CD33 as an independent prognostic marker after adjustment for genetic subgroups [10].

These findings suggest that the prognostic impact of CD33 expression is largely mediated by associated molecular aberrations rather than representing an independent prognostic marker.

Beyond expression levels, genetic variation in CD33, such as the CD33 single-nucleotide polymorphism (SNP) rs12459419 (C>T; Ala14Val), may also influence disease biology and treatment response [12,13]. This SNP, located in exon 2 of the CD33 gene, alters transcript splicing. The T allele promotes skipping of exon 2, resulting in increased expression of a shorter CD33 isoform (D2-CD33) that lacks the extracellular IgV domain required for binding of GO and commonly used diagnostic antibodies. In contrast, the C allele encodes the full-length CD33 protein (FL-CD33) [12]. Available evidence suggests that diagnostic antibodies are limited to detect the CD33 isoform as the epitope detected by the antibody spans the splicing site affected by the CD33 polymorphism [14]. The relationship between rs12459419 and GO response has been investigated extensively. In children, this SNP is associated with lower CD33 expression and with the absence of benefit from adding GO to standard chemotherapy [12]. However, most subsequent studies in adults did not confirm a consistent association between the SNP and clinical outcomes [7,15,16,17,18]. To date, a significant effect has only been demonstrated in *NPM1*-mutated AML, where patients with the CC genotype experienced improved RFS and reduced cumulative incidence of relapse (CIR) when treated with GO in addition to standard induction treatment [19,20]. Findings in other AML subgroups remain contradictory.

Importantly, the impact of the CD33 *rs12459419* genotype on clinical outcomes in adult AML patients in the absence of CD33-targeted therapy remains unclear. In this pre-GO-era study, we aimed to (i) assess the prognostic relevance of the *rs12459419* SNP in adult AML patients receiving chemotherapy alone and (ii) determine whether CD33 expression influences clinical outcomes either across genotype groups or independently of the SNP.

## 2. Results

### 2.1. Baseline Characteristics

A total of 184 patients were included in this study. Patients’ baseline clinical characteristics at the time of initial AML diagnosis are shown in Table 1. Of the 184 patients included, 85 (46%) had the CC genotype, 79 (43%) had the CT genotype and 20 patients (11%) had the TT genotype. The minor allele frequency (MAF) was 32.3%. The median age was 57 years and did not differ between the three groups (*p* = 0.32). There was no statistically significant difference found between the three genotypes in terms of sex, peripheral blood parameters and blasts within bone marrow.

### 2.2. CD33 Expression at First Diagnosis

**Figure 1:**
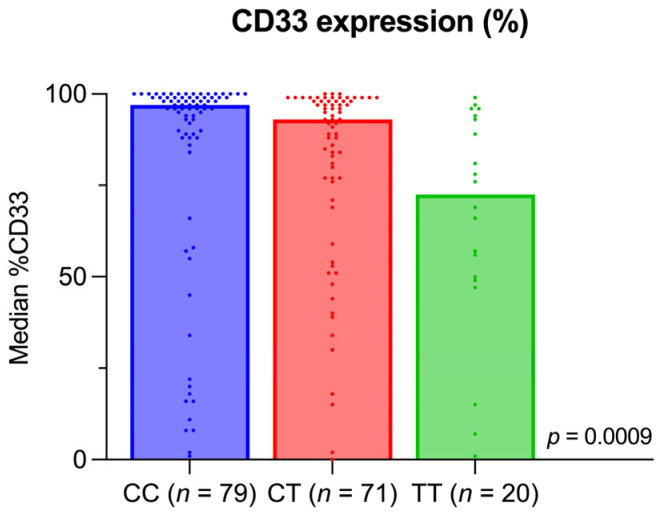
Percentage of CD33-positive blasts at first diagnosis by genotype. Column bars represent median proportion of CD33-positive blasts, with each dot marking an individual value. The full length of CD33 antibody was used.

Data on the percentage of CD33-positive leukemic blasts at initial diagnosis were obtained in 170 of the 184 patients. When analyzing the data using the Kruskal–Wallis test, we found a significant difference between the three genotypes in the percentage of CD33-positive leukemic blasts (***p* = 0.0009**). Using Dunn’s multiple comparisons test as a follow-up test revealed that patients with the CC genotype had a significantly higher percentage of CD33-positive leukemic blasts than those with the TT genotype (***p* = 0.0009**), whilst the difference between CC and CT (*p* = 0.12) was not significant. Comparing CT and TT genotypes, we observed a non-significant trend toward a higher percentage of CD33-positive leukemic blasts in patients with the CT genotype (*p* = 0.06). Further, we observed that the dispersion of the proportion of CD33-positive blasts within genotype groups increases with the presence of one T allele and widens further when two T alleles are present, as shown in Figure 1.

#### 2.2.1. Response to Induction Treatment by Genotype

**Figure A1:**
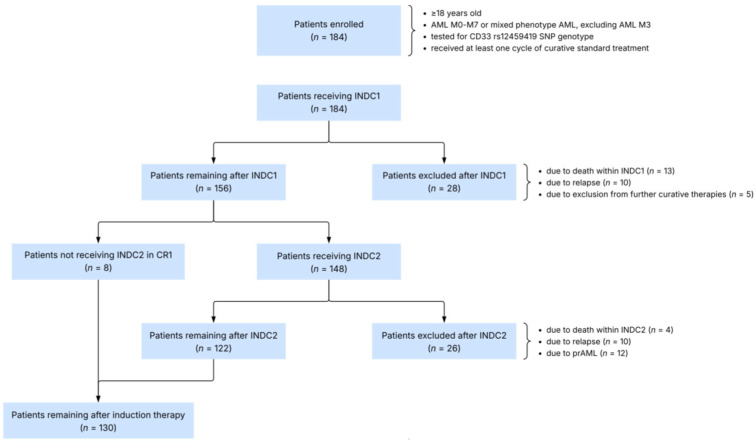
Course of induction treatment.

The course of induction treatment is shown in Figure A1 and listed in Table A1. We compared the three genotypes regarding their response to two cycles of standard induction chemotherapy. Following the first induction cycle (INDC1), 127 of 184 patients (69%) reached a first complete remission (CR1). We found no significant difference in the number of patients in CR1 after INDC1 across the CC, CT, and TT genotypes (*p* = 0.87). A total of 28 patients (15%) were excluded from further response analysis after INDC1; thereof, 46% (*n* = 13) due to death, 36% (*n* = 10) due to relapse, and 18% (*n* = 5) due to exclusion from further curative treatment. A total of 148 of the 156 remaining patients (95%) received the second induction cycle (INDC2); thereof, 131 (89%) achieved or remained in CR1. We did not observe a significant difference between the three genotypes regarding their response to INDC2 (*p* = 0.93). Across both induction cycles, 155 (84%) of all patients enrolled achieved CR1 at some point. We did not observe a significant difference in the number of patients achieving CR1 between the three genotypes (*p* = 0.78). The data can be found in Table 2. If patients remained within CR1 after INDC2, consolidation therapy was initiated. Patients either received autologous HSCT, allogeneic HSCT or consolidation chemotherapy. A total of 13% (*n* = 17) did not have any consolidation treatment, and 3% (*n* = 4) had auto-HSCT followed by allo-HSCT without relapse in between. Maintenance therapy was given to 33 patients (25%) after first-line consolidation or directly after INDC2 (Table A1).

#### 2.2.2. Measurable Residual Disease (MRD) Assessment

We reported the MRD of patients in CR1 after receiving two induction cycles. Patients whose MRD status was monitored by immunophenotyping (IP) did not show a significant difference between the three genotypes (*p* = 0.45). The molecular MRD status of patients with available MRD markers (*n* = 57; NPM1 mutations Type A and B, RUNX1::RUNX1T1, CBFB::MYH11) was assessed via real-time quantitative polymerase chain reaction (RT-qPCR). However, we observed a statistically significant difference in MRD status between genotypes in patients monitored by RT-qPCR (***p* = 0.0097**), with all patients with the CC genotype being MRD-positive in comparison to patients with the CT genotype (71%) and TT genotype (75%).

### 2.3. Outcomes and Survival by Genotype

**Figure 2:**
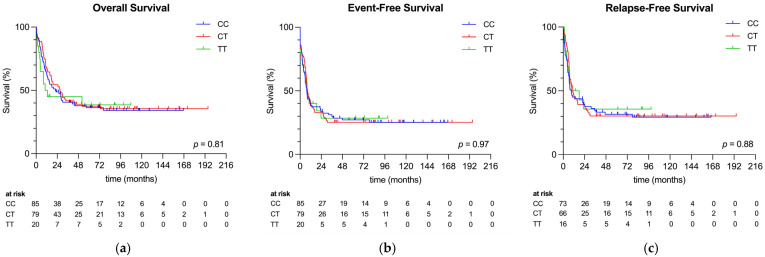
(**a**) Overall survival by genotype; (**b**) event-free survival by genotype; (**c**) relapse-free survival by genotype.

Patients’ outcomes are shown in Table 2, and of all 184 patients included, 115 (63%) died during follow-up. Death rates were similar between patients with CC, CT, and TT genotypes (62% vs. 63% vs. 60%, *p* = 0.96). When analyzing for a difference in the cause of death, we did not find a significant difference between the three genotypes (*p* = 0.054). Nevertheless, it stands out that no patients within the TT genotype died due to therapy-related complications in comparison to the other genotypes. Of the 155 patients achieving CR1, 92 (59%) relapsed. Again, no significant difference was found regarding the percentage of patients experience relapse (*p* = 0.74) across the three genotypes. Although median OS appeared shorter in the TT group compared with the CC and CT groups, this difference was not statistically significant (*p* = 0.81). In contrast, no differences in EFS or RFS were observed between genotypes. The corresponding Kaplan–Meier curves are shown in Figure 2a–c.

### 2.4. Outcomes Depending on CD33 Expression

Additionally, we analyzed differences in OS, EFS, and RFS across the three genotypes between patients with a low percentage of CD33-positive leukemic blasts and patients with a high CD33 percentage. The percentage of CD33-positive leukemic blasts at first diagnosis was obtained in 170 patients. The cut-off value between the two groups was set at 90% CD33-positive blasts; patients were grouped into CD33_low_ (CD33 < 90%) and CD33_high_ (CD33 ≥ 90%). The results are listed in Table 3.

In CD33_high_ patients, there was a trend toward longer OS (*p* = 0.23), EFS (*p* = 0.10), and RFS (*p* = 0.059) in carriers of the T allele, whilst in CD33_low_ patients, carriers of the T allele showed a trend toward shorter survival (OS: *p* = 0.10; EFS: *p* = 0.07; RFS: *p* = 0.09). These observations did not reach significance.

To analyze a linear relationship between survival and ascending percentage of CD33-positive blasts across the three genotypes, we grouped patients according to their genotype into ten groups with increasing percentage of CD33-positive blasts and analyzed the data with the log-rank test for trend. There was no such linear relationship found within OS, EFS, and RFS. The data can be found within the Supplemental Figures S1 and S2.

We further investigated the difference in survival between patients with low and high percentages of CD33-positive blasts, independent of their CD33 rs12459419 SNP genotype. The cut-off value was again set at 90% CD33-positive blasts, and patients were divided into two groups (CD33_low_ < 90% vs. CD33_high_ ≥ 90%). The results are shown in Table 4. There was no difference observed between patients with low versus high percentages of CD33-positive leukemic blasts in OS, EFS, and RFS.

A Cox regression analysis showed that risk stratification according to European LeukemiaNet (ELN) 2022 and age at first diagnosis are significant variables influencing OS, EFS, and RFS. Further, achieving CR1 within INDC1 significantly impacted OS and EFS. Neither the CD33 rs12459419 SNP genotype nor the percentage of CD33-positive leukemic blasts was a predictive factor for survival. The data can be found in Table A2 and Supplemental Table S6.

### 2.5. CC+CT vs. TT

Since we found that two T alleles were associated with a significantly lower percentage of CD33-positive leukemic blasts, we combined patients with the CC and CT genotype and compared them to the TT genotype to further study their prognostic effect. We could not find a statistically significant difference in clinical outcomes and survival between the CC+CT genotypes and the TT genotype. The data can be found within Supplemental Tables S1–S6.

## 3. Discussion

In our cohort, we analyzed the CD33 rs12459419 SNP distribution and its potential association with the proportion of CD33-positive leukemic blasts among all leukemic cells, clinical characteristics, treatment response, and survival in adult AML patients receiving standard induction chemotherapy without GO. The observed genotype distribution and MAF were consistent with previous reports in both adult and pediatric AML populations [12,13,17,18,19]. Notably, the CD33 rs12459419 SNP significantly influenced CD33 expression: patients with the CC genotype exhibited the highest percentage of CD33-positive blasts, followed by CT, and finally TT, which showed the lowest percentage. These findings align with prior studies by Oya et al. [21] and Laszlo et al. [15], who reported a progressive decrease in the percentage of CD33-positive blasts with the presence of one or two T alleles. Although the measurement of CD33 proportion in our study differs from the more commonly applied median CD33 MFI (mean fluorescence intensity) in other reports, existing evidence suggests that the two approaches are broadly comparable [15]. Multiple studies consistently reported the lowest MFI-CD33 expression in patients with the TT genotype and the highest in CC carriers [12,13,15,17], supporting the validity of our results.

Despite these differences observed in the percentage of CD33-positive blasts, baseline patient characteristics—including age, gender, and ELN risk classification—were generally similar across genotypes, which aligns with prior studies [12,13,17].

Based on the observation that patients with the CC or CT genotype generally show a higher proportion of CD33-positive blasts, whereas patients with the TT genotype show a lower percentage in our study, we performed an additional analysis comparing CC+CT versus TT to approximate associations between the percentage of CD33-positive blasts and clinical features. No significant differences were observed apart from the CD33 percentage itself. This contrasts with reports from Pollard et al. [22] and Khan et al. [10], who linked higher CD33 expression to a higher prevalence of FLT3-ITD, NPM1 mutations, trisomy 8, chromosome 11 abnormalities, and intermediate-risk disease, while lower CD33 expression was associated with favorable-risk AML subtypes, including CBF-AML. Similarly, Olombel et al. [23] and other studies [24] reported correlations between high CD33 expression and elevated WBC counts, higher blast proportions in bone marrow, and specific mutational profiles. Differences between our cohort and these prior studies may reflect variations in measurement methods, sample size, and patient selection criteria.

Regarding treatment response and survival, neither the CD33 rs12459419 genotype nor the percentage of CD33-positive blasts influenced the response to standard induction chemotherapy in our study cohort. The only exception in our cohort was the molecular MRD status of patients after receiving two cycles of induction treatment, with all patients of the CC genotype being MRD-positive in comparison to patients of the CT or TT genotype, where some patients reached molecular MRD-negativity. However, the significance of this finding is compromised by the number of patients with missing MRD data included in our analysis.

Our findings fill a gap in the literature, as prior studies predominantly examined outcomes in the context of anti-CD33 immunotherapy, such as gemtuzumab ozogamicin. For example, Gale et al. reported 5-year OS rates in younger adults receiving intensive induction with or without GO, showing modest differences between CC, CT, and TT genotypes even in the no-GO arm [17]. In our adult cohort, 5-year OS and RFS were slightly lower across all genotypes, likely reflecting older patient age and differences in treatment protocols. Cox regression analysis confirmed that age at diagnosis, ELN risk group, and achievement of complete remission within the first induction cycle were independent predictors of survival, whereas the CD33 rs12459419 SNP genotype and the percentage of CD33-positive blasts were not significant predictors in either univariate or multivariate models.

To further explore the potential impact of CD33 expression, we stratified patients by genotype and used a 90% cutoff for CD33-positive leukemic blasts. While no significant differences were observed in OS, EFS, or RFS, a trend toward improved survival for carriers of the T allele was noted within the CD33_high_ subgroup. However, the small number of TT patients limits the interpretability of this observation.

The limitations of our study include the relatively small overall sample size and the very limited number of patients with the TT genotype, which reduces statistical power for subgroup analyses. In addition, patients often received individualized therapy after relapse, including allo-HSCT, which may confound long-term survival outcomes. Additionally, our method of measuring CD33 expression (percentage of CD33-positive blasts among all leukemic cells) differs from the commonly used median MFI in other studies, which may limit direct comparability. Finally, single-center cohort effects and heterogeneity in treatment regimens constrain the generalizability of our findings.

In summary, our findings suggest that patients carrying the CD33 rs12459419 SNP CC and CT genotypes show significantly higher proportions of CD33-positive blasts and may therefore benefit more from CD33-targeted therapies than patients with the TT genotype. However, this potential predictive relevance relates to CD33-targeted therapies and was not assessed in the present study. Given that the lower percentage of CD33-positive leukemic blasts observed in patients with the TT genotype and its potential disadvantage in GO-containing regimens, we propose that testing patients for their SNP genotype prior to administration of targeted treatment could help identify patients more likely to profit from it. Regarding the main question of our study, we did not observe any relevant prognostic impact of either the CD33 rs12459419 SNP or CD33 expression on treatment response, clinical outcomes or survival in adult AML patients undergoing standard induction chemotherapy. To our knowledge, this is the first study evaluating the prognostic impact of the CD33 rs12459419 SNP on outcome in adult AML patients treated with chemotherapy without GO. Further studies with larger patient cohorts are needed to reliably rule out a prognostic role of the CD33 rs12459419 SNP in AML.

## 4. Materials and Methods

### 4.1. Patients

In this retrospective analysis, we investigated 184 adult patients (≥18 years) with AML at first diagnosis who were treated with curative intent and received at least one cycle of standard intensive induction chemotherapy (7+3) at the University Hospital of Bern (Switzerland) between February 2006 and December 2022. Patients with acute promyelocytic leukemia (APL/AML-M3) were excluded from this study. Patients with mixed-phenotype AML were included, provided that myeloid leukemic cells predominated and treatment followed AML-based regimens. All included patients were tested for their CD33 rs12459419 (C>T; Ala14Val) SNP genotype and assigned to their corresponding genotype group. ELN risk assessment was performed based on AML characteristics at initial diagnosis according to the 2022 ELN risk classification [1]. This study was approved by the local ethics committee, Berne, Switzerland (decision number 2024-01251; approval date: 7 August 2024).

### 4.2. CD33 Expression at InitialDiagnosis

CD33 expression at first diagnosis was determined via flow cytometric immunophenotyping using a flow cytometer and a CD33-specific antibody (BD Biosciences, Franklin Lakes, NJ, USA) and reported as the percentage of CD33-positive blasts among all leukemic cells. The p67.6-CD33 antibody was used for analysis, which measures the full-length CD33. According to previous studies [15], the proportion of CD33-positive blasts correlates well with the analysis of median CD33 MFI, which was chosen as a parameter of investigation in other studies from the literature. The percentage of CD33-positive leukemic blasts was assessed primarily in bone marrow samples. In cases where bone marrow aspirates could not be obtained (e.g., punctio sicca), peripheral blood specimens were used for analysis. In 14 patients, no data on the percentage of CD33-positive leukemic blasts at initial diagnosis could be obtained. Therefore, those patients were excluded from analyses involving CD33 expression.

### 4.3. Genotyping of CD33 rs12459419 SNP

To determine the patient’s genotype at position rs12459419 (C>T; Ala14Val) in the CD33 gene, DNA was extracted from peripheral blood mononuclear cells (PBMCs). Amplification of DNA fragments was performed using FIREPol (Solis Biodyne, Tartu, Estonia) and gene-specific primers (X2F 5′-GCCCTGGAAGCTGCTTCCTCA-3′ and X2R 5′-GTGCAGGGCACGAGGACGCA-3′) for exon 2 of the CD33 gene. Sanger sequencing was performed by Microsynth (Balgach, Switzerland).

### 4.4. Definitions

To define outcomes, endpoints, and cut-off values, the 2022 ELN recommendations were considered whilst taking the GraphPad Prism Statistics Guide into account [1,25].

Complete remission (CR) was achieved when bone marrow samples contained fewer than 5% leukemic blasts, and no evidence of extramedullary AML was present. Patients failing attainment of CR1 by the end of two induction cycles were classified as having primary refractory AML (prAML). Relapse included not only morphological but also molecular and MRD relapse as well. In survival analysis, patients were censored at the cut-off date if none of the predefined endpoints were reached by then. Patients lost to follow-up were censored on the date they were last known to be alive. Median survival was defined as the point in time when either event, relapse or death has occurred in 50% of the patients. If this did not occur by the cut-off date, the median survival was reported as “undefined”. Data were collected until 1 February 2023, which served as the cut-off date for this study.

OS was calculated from the date of first diagnosis until the occurrence of death or—if alive—until censored. EFS was calculated from the date of first diagnosis until the occurrence of death, relapse or censoring, while deaths without a relapse were considered competing events. If prAML was present or if patients died without response assessment, EFS was zero days. RFS was calculated from the date of achieving CR1 within induction therapy until relapse, death or censoring, while death without a relapse was considered a competing event. Fatalities occurring within 30 days of the start of INDC1 or INDC2 were classified as early death. Casualties within 40 days after beginning curative intended treatment or casualties due to graft-versus-host disease at any time following allo-HSCT were categorized as treatment-related mortality (TRM). Unexpected and sudden casualties not attributed to complications of AML therapy, as well as patients who died from progressive disease, were excluded from TRM.

### 4.5. MRD Assessment

For MRD assessment, bone marrow samples of patients within CR1 after INDC2 were analyzed. NPM1 mutations of the types A or B, RUNX1::RUNX1T1-fusion gene and CBFB::MYH11-fusion gene were considered molecular MRD markers. If molecular MRD markers were detected at first diagnosis, patients were monitored for MRD using RT-qPCR as well as IP, where cells with leukemia-associated immunophenotypes (LAIPs) were quantified. If no molecular MRD markers were present at first diagnosis, patients were monitored for MRD using IP only. MRD-negativity was considered as LAIPs were <0.1% or if the MRD-marker/ABL1-ratio was <0.0001 and MRD markers were not detectable.

### 4.6. Statistical Analysis

To assess differences across the three genotype groups, the Kruskal–Wallis test was used if variables were continuous. If variables were categorical, the Chi-square test or Fisher’s exact test was applied, whilst the latter was used if Chi-square calculations were invalid due to data distribution. Survivals were estimated using the Kaplan–Meier method and compared across the three genotypes using the log-rank (Mantel–Cox) test or log-rank test for trend. Median follow-up was estimated using the reverse Kaplan–Meier method. *p*-values below 0.05 were considered statistically significant. Statistical analysis was performed using GraphPad Prism version 10.6.0 for macOS (GraphPad Software, Boston, MA, USA, www.graphpad.com) and R (Version 4.5.1, R Foundation for Statistical Computing, Vienna, Austria) for the Cox-proportional hazard model.

## Figures and Tables

**Table 1:** Patient characteristics at initial diagnosis.

Parameter	CC (*n* = 85)	CT (*n* = 79)	TT (*n* = 20)	All Patients (*n* = 184)	*p*-Value
Male, *n* (%)	46 (54)	42 (53)	12 (60)	100 (54)	0.85
Age, median (range)	59 (19–73)	54 (23–75)	60 (45–71)	57 (19–75)	0.32
Hemoglobin g/L, median (range)	88 (38–137)	86 (37–151)	86 (43–113)	87 (37–151)	0.49
Leukocytes G/L, median (range)	15 (0–181)	12 (1–575)	42 (1–303)	15 (0–575)	0.30
Platelets G/L, median (range)	71 (6–714)	66 (7–405)	69 (9–242)	70 (6–714)	0.38
LDH U/L, median (range)	766 (156–7108)	824 (151–18,856)	1150 (276–3552)	844 (151–18,856)	0.12
Blasts PB %, median (range)	46 (0–97)	40 (0–99)	51 (2–90)	45 (0–99)	0.58
Blasts BM %, median (range) ^a^	80 (5–97)	80 (15–95)	75 (20–95)	80 (5–97)	0.71
% CD33-positive leukemic blasts, median (range) ^b^	97 (1–100)	93 (0–100)	73 (1–99)	95 (0–100)	**0.0009**
FAB classification, *n* (%)					
M0	8 (9)	9 (11)	0 (0)	17 (9)	
M1 ^c^	25 (29)	19 (24)	7 (35)	51 (28)	
M2	18 (21)	22 (28)	5 (25)	45 (24)	
M4	23 (27)	13 (16)	5 (25)	41 (22)	
M5	7 (8)	10 (13)	3 (15)	20 (11)	
M6	3 (4)	3 (4)	0 (0)	6 (3)	
M7	0 (0)	1 (1)	0 (0)	1 (1)	
Mixed (B/myeloid)	1 (1)	1 (1)	0 (0)	2 (1)	
Mixed (T/myeloid)	0 (0)	1 (1)	0 (0)	1 (1)	
De novo AML, *n* (%)	66 (78)	60 (76)	13 (65)	139 (76)	0.58
sAML (MDS/MPN-related), *n* (%)	16 (19)	14 (18)	5 (25)	35 (19)	
tAML (therapy-related), *n* (%)	3 (4)	5 (6)	2 (10)	10 (5)	
Cytogenetic aberrations, *n* (%) ^d^					0.19
Normal karyotype	45 (54)	35 (45)	10 (50)	90 (50)	
Complex karyotype	11 (13)	10 (13)	6 (30)	27 (15)	
Abnormal karyotype	27 (33)	32 (42)	4 (20)	63 (35)	
*t(8;21)(q22;q22)*	3 (11)	5 (16)	1 (25)	9 (14)	
*inv(16)(p13q22)/t(16;16)(p13;q22)*	2 (7)	5 (16)	0 (0)	7 (11)	
*t(9;11)(p21;q23)*	1 (4)	0 (0)	1 (25)	2 (3)	
*t(v;11q23)*	2 (7)	6 (19)	0 (0)	8 (13)	
*inv(3)(q21q26)/t(3;3)(q21;q26)*	1 (4)	1 (3)	0 (0)	2 (3)	
*t(9;22)(q34;q11)*	1 (4)	1 (3)	0 (0)	2 (3)	
*t(6;9)(p23;q34)*	0 (0)	1 (3)	0 (0)	1 (2)	
*Other*	17 (63)	13 (41)	2 (50)	32 (51)	
AML-associated mutations, *n* (%)					
*NPM1_mut_ without FLT3-ITD*	15 (18)	14 (18)	4 (20)	33 (18)	
*NPM1_wt_ with FLT3-ITD*	6 (7)	9 (11)	2 (10)	17 (9)	
*NPM1_mut_ with FLT3-ITD*	15 (18)	10 (13)	4 (20)	29 (16)	
*CEBPA_dm_*	1 (1)	2 (3)	0 (0)	3 (2)	
*TP53_mut_*	3 (4)	1 (1)	0 (0)	4 (2)	
*RUNX1_mut_*	2 (2)	3 (4)	2 (10)	7 (4)	
Other	43 (51)	40 (51) ^e^	8 (40) ^f^	91 (49)	
ELN-risk classification (2022), *n* (%)					0.051
Favorable	21 (25)	27 (34)	4 (20)	52 (28)	
Intermediate	39 (46)	19 (24)	9 (45)	67 (36)	
Adverse	25 (29)	33 (42)	7 (35)	65 (35)	

BM: bone marrow; dm: double mutated; ELN: European LeukemiaNet; FAB: French–American–British; LDH: lactate dehydrogenase; MDS: myelodysplastic syndrome; MPN: myeloproliferative neoplasm; mut: mutated; PB: peripheral blood; wt: wildtype; ^a^ one value each is missing in the CC group and CT group; ^b^ six values are missing in the CC group, and eight values are missing in the CT group; ^c^ includes AML-M1, AML not further classifiable, AML from MDS and AML from MPN); ^d^ two cytogenetics each are missing in the CC group and CT group; ^e^ one patient had *NPM1_mut_*, *RUNX1_mut_* and *FLT3-ITD*; ^f^ one patient had *CEBPA_dm_* with *FLT3-ITD*; percentages may not total 100 due to rounding. Values with *p* < 0.05 are marked in bold.

**Table 2:** Outcomes and survival by genotype.

Outcomes and Survival	CC (*n* = 85)	CT (*n* = 79)	TT (*n* = 20)	All Patients (*n* = 184)	*p*-Value
Death from any cause, *n* (%)	53 (62)	50 (63)	12 (60)	115 (63)	0.96
Treatment-related mortality	9 (17)	11 (22)	0 (0)	20 (17)	0.054
Death due to progression of AML	34 (64)	37 (74)	11 (92)	82 (71)	
Other causes of death	10 (19)	2 (4)	1 (8)	13 (11)	
Median OS, months	22.5	27.1	11.5	25.8	0.81
Median follow-up OS, months	83.4	91.9	88.8	88.3	0.15
Patients that reached CR1, *n* (%)	73 (86)	66 (84)	16 (80)	155 (84)	0.78
Relapse, *n* (%)	41 (56)	41 (62)	10 (63)	92 (59)	0.74
Allo-HSCT after relapse, *n* (%)	16 (39)	15 (37)	3 (30)	34 (37)	
Death after allo-HSCT as relapse treatment, *n* (%)	8 (50)	8 (53)	1 (33)	17 (50)	
Median RFS, months ^a^	8.9	10.8	13.5	9.9	0.88
Median follow-up RFS, months	86.1	101.5	87.9	89.0	0.10
Median EFS, months	7.6	8.5	8.1	8.0	0.97
Median follow-up EFS, months	88.3	102.1	88.8	89.7	0.12

Allo-HSCT: allogeneic hematopoietic stem cell transplantation; CR1: first complete remission; EFS: event-free survival; OS: overall survival; RFS: relapse-free survival; ^a^ RFS was only reported for patients who reached CR1 within induction cycle 1 and/or induction cycle 2; percentages may not total 100 due to rounding.

**Table 3:** Survival by genotype depending on the percentage of CD33-positive leukemic blasts.

Survival	CC (*n* = 79)	CT (*n* = 71)	TT (*n* = 20)	All Patients ^a^ (*n* = 170)	*p*-Value
Median OS, months	25.8	26.9	11.5	26.2	0.86
Median OS of CD33_low_ (<90%), months	57.1 (*n* = 23)	17.4 (*n* = 32)	8.3 (*n* = 14)	19.2 (*n* = 69)	0.10
Median OS of CD33_high_ (≥90%), months	15.4 (*n* = 56)	32.5 (*n* = 39)	u.d. (*n* = 6)	27.9 (*n* = 101)	0.23
Median EFS, months	7.6	8.6	8.1	8.2	0.99
Median EFS of CD33_low_ (<90%), months	25.8 (*n* = 23)	7.7 (*n* = 32)	6.2 (*n* = 14)	7.9 (*n* = 69)	0.07
Median EFS of CD33_high_ (≥90%), months	7.1 (*n* = 56)	13.5 (*n* = 39)	54.2 (*n* = 6)	8.2 (*n* = 101)	0.10
Median RFS, months	9.3 (*n* = 69)	10.8 (*n* = 60)	13.5 (*n* = 16)	10.4 (*n* = 145)	0.92
Median RFS of CD33_low_ (<90%), months	96.6 (*n* = 20)	7.1 (*n* = 26)	6.0 (*n* = 11)	9.5 (*n* = 57)	0.09
Median RFS of CD33_high_ (≥90%), months	6.8 (*n* = 49)	20.0 (*n* = 34)	u.d. (*n* = 5)	10.4 (*n* = 88)	0.059

EFS: event-free survival; OS: overall survival; RFS: relapse-free survival; u.d.: undefined; ^a^ 14 patients were excluded from analysis due to missing data on the percentage of CD33-positive leukemic blasts.

**Table 4:** Survival CD33_low_ vs. CD33_high_ [90%] ^a^.

Survival	CD33_low_ (<90%) (*n* = 69)	CD33_high_ (≥90%) (*n* = 101)	*p*-Value
Median OS, months	19.2	27.9	0.48
Median EFS, months	7.9	8.2	0.76
Median RFS, months	9.5	10.4	0.91

EFS: event-free survival; OS: overall survival; RFS: relapse-free survival; ^a^ 14 patients were excluded from analysis due to missing data on the percentage of CD33-positive leukemic blasts.

## Data Availability

All primary data can be provided upon request.

## Supplementary Materials

The following supporting information can be downloaded at: https://www.mdpi.com/article/10.3390/ijms27094050/s1.

## Appendix A

**Table A1:** Response to induction treatment.

Parameter	CC (*n* = 85)	CT (*n* = 79)	TT (*n* = 20)	All Patients (*n* = 184)	*p*-Value
Induction therapy—INDC1, *n* (%)	85 (100)	79 (100)	20 (100)	184 (100)	
CR1 after INDC1	60 (71)	54 (68)	13 (65)	127 (69)	0.87
No CR1 after INDC1 ^a^	25 (29)	25 (32)	7 (35)	57 (31)	
Patients excluded after INDC1, *n* (%)	16 (19)	10 (13)	2 (10)	28 (15)	
Due to relapse	7 (44)	3 (30)	0 (0)	10 (36)	
Due to death from any cause	6 (38)	5 (50)	2 (100)	13 (46)	
Thereof early death	5 (83)	5 (100)	2 (100)	12 (92)	
No further curative therapies	3 (19)	2 (20)	0 (0)	5 (18)	
Patients remaining after INDC1, *n* (%)	69 (81)	69 (87)	18 (90)	156 (85)	
Patients who have received INDC2	67 (97)	64 (93)	17 (94)	148 (95)	
Patients in CR1 without INDC2 ^b^	2 (3)	5 (7)	1 (6)	8 (5)	
Induction therapy—INDC2, *n* (%)	67 (79)	64 (81)	17 (85)	148 (80)	
Intensive chemotherapy ^c^	67 (100)	62 (97)	16 (94)	145 (98)	
Non-intensive chemotherapy ^d^	0 (0)	2 (3)	1 (6)	3 (2)	
CR1 after INDC2	60 (90)	56 (88)	15 (88)	131 (89)	0.93
No CR1 after INDC2 ^a,e^	7 (10)	8 (13)	2 (12)	17 (11)	
Patients MRD status after INDC2, *n* (%)					
Patients with IP-Monitoring	35 (100)	32 (100)	7 (100)	74 (100)	0.45
MRD_positive_ (IP)	1 (3)	4 (13)	1 (14)	6 (8)	
MRD_negative_ (IP)	14 (40)	9 (28)	2 (29)	25 (34)	
IP missing	20 (57)	19 (59)	4 (57)	43 (58)	
Patients with RT-qPCR-Monitoring	25 (100)	24 (100)	8 (100)	57 (100)	**0.0097**
MRD_positive_ (RT-qPCR)	25 (100)	17 (71)	6 (75)	48 (84)	
MRD_negative_ (RT-qPCR)	0 (0)	1 (4)	1 (13)	2 (4)	
RT-qPCR missing	0 (0)	6 (25)	1 (13)	7 (12)	
Patients excluded after INDC2, *n* (%)	13 (19)	9 (14)	4 (24)	26 (18)	
Due to relapse	6 (46)	2 (22)	2 (50)	10 (38)	
Due to death from any cause	2 (15)	1 (11)	1 (25)	4 (15)	
Thereof early death	1 (50)	1 (100)	1 (100)	3 (75)	
Due to prAML	5 (38)	6 (67)	1 (25)	12 (46)	
Patients remaining after INDC2, *n* (%)	56 (84)	60 (94)	14 (82)	130 (88)	
Patients in CR1 after INDC2	54 (96)	55 (92)	13 (93)	122 (94)	
Patients in CR1 without INDC2	2 (4)	5 (8)	1 (7)	8 (6)	
Consolidation therapy, *n* (%)	56 (100)	60 (100)	14 (100)	130 (100)	
Autologous HSCT (auto-HSCT)	35 (63) ^f^	33 (55) ^g^	9 (64)	77 (59)	
Allogeneic HSCT (allo-HSCT)	6 (11)	17 (28)	2 (14)	25 (19)	
Auto-HSCT and allo-HSCT without relapse	2 (4) ^h^	2 (3)	0 (0)	4 (3)	
Consolidation chemotherapy only	5 (9)	2 (3)	0 (0)	7 (5)	
No post-remission therapy	8 (14)	6 (10)	3 (21)	17 (13)	
Maintenance therapy, *n* (%) ^i^	16 (29)	13 (22)	4 (29)	33 (25)	

CR1: first complete remission; HSCT: hematopoietic stem cell transplantation; INDC1: induction cycle 1; INDC2: induction cycle 2; IP: immunophenotyping by flow cytometry; MRD: measurable residual disease; prAML: primary refractory AML; RT-qPCR: realtime-quantitative-PCR; ^a^ including patients who died before response assessment could be performed; ^b^ patients in CR1 who did not receive INDC2 and will be again included in the post-remission therapy calculations of this table; ^c^ patients in general were given anthracycline + cytarabine (<60 years) or cytarabine only (≥60 years); ^d^ includes the following drugs: decitabine, hydroxyurea and sorafenib; ^e^ including three patients who showed a relapse on the day of bone marrow assessment; ^f^ one patient received auto-HSCT and consolidation chemotherapy; ^g^ one patient received auto-HSCT and consolidation chemotherapy; ^h^ one patient received allo-HSCT, auto-HSCT and consolidation chemotherapy without relapse; ^i^ includes the following drugs and treatments: azacitidine, decitabine, donor lymphocyte infusion, enasidenib, gilteritinib, imatinib, lenalidomide, midostaurin, sorafenib and venetoclax; percentages may not total 100 due to rounding. Values with *p* < 0.05 are marked in bold.

**Table A2:** Univariable and multivariable model of OS, EFS, and RFS.

				Univariable Model					
	Overall Survival			Event-Free Survival			Relapse-Free Survival		
Characteristic	HR	95% CI	*p*-Value	HR	95% CI	*p*-Value	HR	95% CI	*p*-Value
CD33 SNP genotype									
CC	-	-		-	-		-	-	
CT	0.92	0.63, 1.36	0.70	0.97	0.68, 1.39	0.90	0.92	0.62, 1.38	0.70
TT	1.12	0.60, 2.09	0.70	0.96	0.54, 1.72	0.90	0.87	0.44, 1.72	0.70
% CD33-positive leukemic blasts	1.00	1.00, 1.01	0.70	1.00	1.0, 1.00	0.90	1.00	0.99, 1.01	>0.90
ELN risk stratification (2022)									
Favorable	-	-		-	-		-	-	
Intermediate	2.11	1.25, 3.56	**0.005**	2.18	1.37, 3.48	**0.001**	2.28	1.39, 3.76	**0.001**
Adverse	2.85	1.71, 4.76	**<0.001**	2.56	1.61, 4.06	**<0.001**	2.20	1.32, 3.68	**0.003**
Sex									
Female	-	-		-	-		-	-	
Male	1.50	1.03, 2.19	**0.034**	1.69	1.19, 2.39	**0.003**	1.66	1.13, 2.46	**0.010**
Age at first diagnosis	1.03	1.01, 1.04	**0.003**	1.03	1.01, 1.04	**<0.001**	1.02	1.00, 1.04	**0.022**
CR after INDC1									
No	-	-		-	-		-	-	
Yes	0.40	0.27, 0.58	**<0.001**	0.36	0.25, 0.51	**<0.001**	0.67	0.42, 1.07	0.10
				**Multivariable Model**					
	**Overall Survival**			**Event-Free Survival**			**Relapse-Free Survival**		
**Characteristic**	**HR**	**95% CI**	***p*-Value**	**HR**	**95% CI**	***p*-Value**	**HR**	**95% CI**	***p*-Value**
CD33 SNP genotype									
CC	-	-		-	-		-	-	
CT	0.93	0.63, 1.37	0.70	1.02	0.71, 1.46	>0.90	1.01	0.67, 1.52	>0.90
TT	1.16	0.61, 2.19	0.60	0.87	0.49, 1.57	0.60	0.78	0.39, 1.56	0.50
% CD33-positive leukemic blasts	1.00	1.00, 1.01	0.20	1.00	1.00, 1.01	0.70	1.00	0.99, 1.01	0.80
ELN risk stratification (2022)									
Favorable	-	-		-	-		-	-	
Intermediate	1.69	0.98, 2.90	0.059	1.85	1.14, 3.02	**0.013**	2.13	1.26, 3.59	**0.005**
Adverse	2.16	1.25, 3.74	**0.006**	1.97	1.21, 3.22	**0.007**	2.06	1.20, 3.55	**0.009**
Sex									
Female	-	-		-	-		-	-	
Male	1.15	0.78, 1.70	0.50	1.29	0.90, 1.85	0.20	1.36	0.90, 2.03	0.14
Age at first diagnosis	1.02	1.00, 1.04	0.059	1.02	1.00, 1.04	**0.025**	1.02	1.00, 1.04	0.052
CR after INDC1									
No	-	-		-	-		-	-	
Yes	0.54	0.36, 0.81	**0.003**	0.48	0.33, 0.71	**<0.001**	0.92	0.56, 1.51	0.70

CI: confidence interval; CR: complete remission; ELN: European LeukemiaNet; HR: hazard ratio; INDC1: induction cycle 1; SNP: single-nucleotide polymorphism. Values with *p* < 0.05 are marked in bold.
