# Supplementary material for: Analysis of Clinical Impact of CD33 rs12459419 Single-Nucleotide Polymorphism in AML Treated with Intensive Chemotherapy Without Gemtuzumab Ozogamicin
Source: Int J Mol Sci. 2026 Apr 30;27(9):4050. doi: 10.3390/ijms27094050 (PMC13163365; doi:10.3390/ijms27094050)
Supplement: Supplementary file 1 [file ijms-27-04050-s001.zip › ijms-4271859-supplementary.pdf]

Supplementary Materials

Analysis of Clinical Impact of CD33 rs12459419 Single Nucleotide Polymorphism in AML Treated with Intensive Chemotherapy Without Gemtuzumab Ozogamicin

Sophie Helfenstein, Inna Shaforostova, Katja Seipel, Marie-Noelle Kronig, Myriam Legros, Ulrike Bacher, Thomas Pabst

Supplemental Figure S1. Flow cytometry gating strategy for CD33 analysis

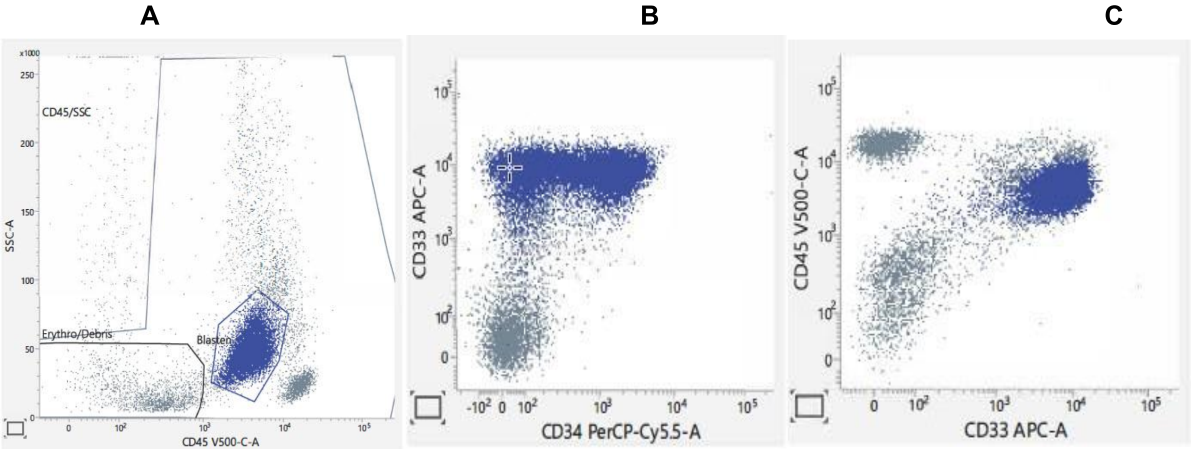

A: CD45 vs. SSC-A gating
B: CD45 vs. CD33 gating
C: CD34 vs. CD33 gating

Supplemental Figure S2. Effect of the percentage of CD33-positive leukemic blasts on survival (CC vs CT vs TT). A) Clustered bar plot of OS across CD33 expression levels stratified by CD33 SNP genotype

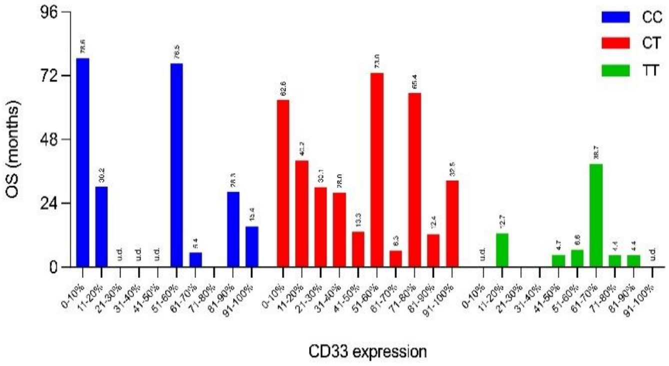

B) Clustered bar plot of RFS across CD33 expression levels stratified by CD33 SNP genotype

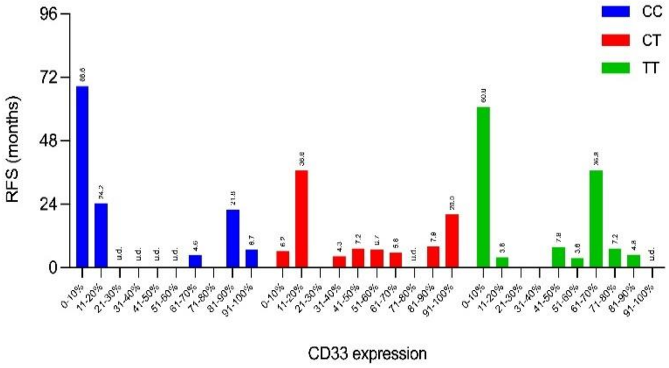

C) B) Clustered bar plot of EFS across CD33 expression levels stratified by CD33 SNP genotype

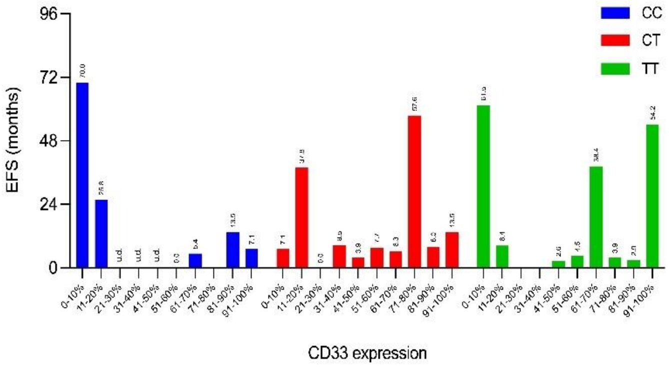

EFS: event-free survival; *n*: number of patients; OS: overall survival; RFS: relapse-free survival; u.d.: undefined.

Supplemental Table S1. Patient characteristics at initial diagnosis (CC+CT vs TT).

**Parameter**	**CC+CT** (*n* = 164)	**TT** (*n* = 20)	**All patients** (*n* = 184)	***p*-value**
Male, *n* (%)	88 (54)	12 (60)	100 (54)	0.64
Age, median (range)	56 (19-75)	60 (45-71)	57 (19-75)	0.31
Hemoglobin g/l, median (range)	87 (37-151)	86 (43-113)	87 (37-151)	0.40
Leukocytes G/l, median (range)	14 (0-575)	42 (1-303)	15 (0-575)	0.13
Platelets G/l, median (range)	70 (6-714)	69 (9-242)	70 (6-714)	0.85
LDH U/l, median (range)	787 (151-18856)	1150 (276-3552)	844 (151-18856)	0.06
Blasts PB %, median (range)	45 (0-99)	51 (2-90)	45 (0-99)	0.46
Blasts BM %, median (range)	80 (5-97) [a]	75 (20-95)	80 (5-97)	0.55
% CD33-positive leukemic blasts, median (range)	96 (0-100) [b]	73 (1-99)	95 (0-100)	**0.0013**
**FAB classification, *n* (%)**				
M0	17 (10)	0 (0)	17 (9)	
M1 [c]	44 (27)	7 (35)	51 (28)	
M2	40 (24)	5 (25)	45 (24)	
M4	36 (22)	5 (25)	41 (22)	
M5	17 (10)	3 (15)	20 (11)	
M6	6 (4)	0 (0)	6 (3)	
M7	1 (1)	0 (0)	1 (1)	
Mixed (B/myeloid)	2 (1)	0 (0)	2 (1)	
Mixed (T/myeloid)	1 (1)	0 (0)	1 (1)	
De novo AML, *n* (%)	126 (77)	13 (65)	139 (76)	0.28
sAML (MDS/MPN-related), *n* (%)	30 (18)	5 (25)	35 (19)	
tAML (therapy-related), *n* (%)	8 (5)	2 (10)	10 (5)	
**Cytogenetic aberrations, *n* (%)** [d]				0.09
Normal karyotype	80 (50)	10 (50)	90 (50)	
Complex karyotype	21 (13)	6 (30)	27 (15)	
Abnormal karyotype	59 (37)	4 (20)	63 (35)	
t(8;21)(q22;q22)	8 (5)	1 (5)	9 (5)	
inv(16)(p13q22) or t(16;16)(p13;q22)	7 (4)	0 (0)	7 (4)	
t(9;11)(p21;q23)	1 (1)	1 (5)	2 (1)	
t(v;11q23)	8 (5)	0 (0)	8 (4)	
inv(3)(q21q26) or t(3;3)(q21;q26)	2 (1)	0 (0)	2 (1)	
t(9;22)(q34;q11)	2 (1)	0 (0)	2 (1)	
t(6;9)(p23;q34)	1 (1)	0 (0)	1 (1)	
Other	30 (19)	2 (10)	32 (18)	
**AML-associated mutations, *n* (%)**				
$NPM1_{mut}$ without FLT3-ITD	29 (18)	4 (20)	33 (18)	
$NPM1_{wt}$ with FLT3-ITD	15 (9)	2 (10)	17 (9)	
$NPM1_{mut}$ with FLT3-ITD	25 (15)	4 (20)	29 (16)	
$CEBPA_{dm}$	3 (2)	0 (0)	3 (2)	
$TP53_{mut}$	4 (2)	0 (0)	4 (2)	
$RUNX1_{mut}$	5 (3)	2 (10)	7 (4)	
Other	83 (51) [e]	8 (40) [f]	91 (49)	
**ELN-risk classification, *n* (%)**				0.63
Favorable	48 (29)	4 (20)	52 (28)	
Intermediate	58 (35)	9 (45)	67 (36)	
Adverse	58 (35)	7 (35)	65 (35)	

BM: bone marrow; dm: double mutated; ELN: European LeukemiaNet; FAB: French-American-British; LDH: lactate dehydrogenase; MDS: myelodysplastic syndrome; MPN: myeloproliferative neoplasm; mut: mutated; PB: peripheral blood; wt: wildtype; [a] two values are missing in the CC+CT group; [b] 14 values are missing in the CC+CT group; [c] includes: AML-M1, AML not further classifiable, AML from MDS (WHO 2008) and AML from MPN (WHO 2008); [d] four cytogenetics are missing in the CC+CT group; [e] one patient had $NPM1_{mut}$, $RUNX1_{mut}$ and FLT3-ITD; [f] one patient had $CEBPA_{dm}$ with FLT3-ITD; percentages may not total 100 due to rounding.

Supplemental Table S2. Response to induction treatment (CC+CT vs TT).

Parameter	CC+CT (*n* = 164)	TT (*n* = 20)	All patients (*n* = 184)	*p*-value
Induction therapy – INDC1, *n* (%)	164 (100)	20 (100)	184 (100)	
CR1 after INDC1	114 (70)	13 (65)	127 (69)	0.79
No CR1 after INDC1 [a]	50 (30)	7 (35)	57 (31)	
Patients excluded after INDC1, *n* (%)	26 (16)	2 (10)	28 (15)	
Due to relapse	10 (38)	0 (0)	10 (36)	
Due to death from any cause	11 (42)	2 (100)	13 (46)	
Thereof early death	10 (91)	2 (100)	12 (92)	
No further curative therapies	5 (19)	0 (0)	5 (18)	
Patients remaining after INDC1, *n* (%)	138 (84)	18 (90)	156 (85)	
Patients who have received INDC2	131 (95)	17 (94)	148 (95)	
Patients in CR1 without INDC2 [b]	7 (5)	1 (6)	8 (5)	
Induction therapy – INDC2, *n* (%)	131 (80)	17 (85)	148 (80)	
Intensive chemotherapy [c]	129 (98)	16 (94)	145 (98)	
Non-intensive chemotherapy [d]	2 (2)	1 (6)	3 (2)	
CR1 after INDC2	116 (89)	15 (88)	131 (89)	>0.99
No CR1 after INDC2 [a,e]	15 (11)	2 (12)	17 (11)	
Patients MRD status after INDC2, *n* (%)				
Patients with IP-Monitoring	67 (100)	7 (100)	74 (100)	0.68
$MRD_{positive}$ (IP)	5 (7)	1 (14)	6 (8)	
$MRD_{negative}$ (IP)	23 (34)	2 (29)	25 (34)	
IP missing	39 (58)	4 (57)	43 (58)	
Patients with RT-qPCR-Monitoring	49 (100)	8 (100)	57 (100)	0.30
$MRD_{positive}$ (RT-qPCR)	42 (86)	6 (75)	48 (84)	
$MRD_{negative}$ (RT-qPCR)	1 (2)	1 (13)	2 (4)	
RT-qPCR missing	6 (12)	1 (13)	7 (12)	
Patients excluded after INDC2, *n* (%)	22 (17)	4 (24)	26 (18)	
Due to relapse	8 (36)	2 (50)	10 (38)	
Due to death from any cause	3 (14)	1 (25)	4 (15)	
Thereof early death	2 (67)	1 (100)	3 (75)	
Due to prAML	11 (50)	1 (25)	12 (46)	
Patients remaining after INDC2, *n* (%)	116 (89)	14 (82)	130 (88)	
Patients in CR1 after INDC2	109 (94)	13 (93)	122 (94)	
Patients in CR1 without INDC2	7 (6)	1 (7)	8 (6)	
Consolidation therapy, *n* (%)	116 (100)	14 (100)	130 (100)	
Autologous HSCT (auto-HSCT)	68 (59) [f]	9 (64)	77 (59)	
Allogeneic HSCT (allo-HSCT)	23 (20)	2 (14)	25 (19)	
Auto-HSCT and allo-HSCT without relapse	4 (3) [g]	0 (0)	4 (3)	
Consolidation chemotherapy only	7 (6)	0 (0)	7 (5)	
No post-remission therapy	14 (12)	3 (21)	17 (13)	
Maintenance therapy, *n* (%) [h]	29 (25)	4 (29)	33 (25)	

CR1: first complete remission; HSCT: hematopoietic stem cell transplantation; INDC1: induction cycle 1; INDC2: induction cycle 2; IP: immunophenotyping by flow cytometry; MRD: measurable residual disease; prAML: primary refractory AML; RT-qPCR: realtime-quantitative-PCR; [a] including patients that died before response assessment could be performed; [b] patients in CR1 that did not receive INDC2 and will be again included in the post-remission therapy calculations of this table; [c] patients in general were given anthracycline + cytarabine (<60 years) or cytarabine only (≥60 years); [d] includes the following drugs: decitabine, hydroxyurea and sorafenib; [e] including three patients who showed a relapse on the day of bone marrow assessment; [f] two patients received auto-HSCT and consolidation chemotherapy; [g] one patient received allo-HSCT, auto-HSCT and consolidation chemotherapy without relapse; [h] includes the following drugs and treatments: azacitidine, decitabine, donor lymphocyte infusion, enasidenib, gilteritinib, imatinib, lenalidomide, midostaurin, sorafenib and venetoclax; percentages may not total 100 due to rounding.

Supplemental Table S3. Outcomes and survival by genotype (CC+CT vs TT).

**Outcomes and survival**	**CC+CT** (*n* = 164)	**TT** (*n* = 20)	**All patients** (*n* = 184)	***p*-value**
Death from any cause, *n* (%)	103 (63)	12 (60)	115 (63)	0.81
Treatment-related mortality	20 (19)	0 (0)	20 (17)	0.22
Death due to progression of AML	71 (69)	11 (92)	82 (71)	
Other causes of death	12 (12)	1 (8)	13 (11)	
Median OS, months	26.2	11.5	25.8	0.61
Median follow-up OS, months	88.3	88.8	88.3	0.10
Patients that reached CR1, *n* (%)	139 (85)	16 (80)	155 (84)	0.52
Relapse, *n* (%)	82 (59)	10 (63)	92 (59)	>0.99
Allo-HSCT after relapse, *n* (%)	31 (38)	3 (30)	34 (37)	
Death after allo-HSCT as relapse treatment, *n* (%)	16 (52)	1 (33)	17 (50)	
Median RFS, months [a]	9.9	13.5	9.9	0.77
Median follow-up RFS, months	91.2	87.9	89.0	0.06
Median EFS, months	8.0	8.1	8.0	0.91
Median follow-up EFS, months	91.9	88.8	89.7	0.07

Allo-HSCT: allogeneic hematopoietic stem cell transplantation; CR1: first complete remission; EFS: event-free survival; OS: overall survival; RFS: relapse-free survival; [a] RFS was only reported for patients that reached CR1 within induction cycle 1 and/or induction cycle 2; percentages may not total 100 due to rounding.

Supplemental Table S4. Survival by genotype depending on the percentage of CD33-positive leukemic blasts (CC+CT vs TT).

**Survival**	**CC+CT** (n = 150)	**TT** (n = 20)	**All patients** [a] (n = 170)	***p*-value**
Median OS, months	26.9	11.5	26.2	0.59
Median OS of $CD33_{low}$ (<90%), months	26.9 (n = 55)	8.3 (n = 14)	19.2 (n = 69)	0.08
Median OS of $CD33_{high}$ (≥90%), months	25.8 (n = 95)	u.d. (n = 6)	27.9 (n = 101)	0.21
Median EFS, months	8.2	8.1	8.2	0.99
Median EFS of $CD33_{low}$ (<90%), months	8.6 (n = 55)	6.2 (n = 14)	7.9 (n = 69)	0.32
Median EFS of $CD33_{high}$ (≥90%), months	8.0 (n = 95)	54.2 (n = 6)	8.2 (n = 101)	0.23
Median RFS, months	10.4 (n = 129)	13.5 (n = 16)	10.4 (n = 145)	0.79
Median RFS of $CD33_{low}$ (<90%), months	10.8 (n = 46)	6.0 (n = 11)	9.5 (n = 57)	0.35
Median RFS of $CD33_{high}$ (≥90%), months	9.9 (n = 83)	u.d. (n = 5)	10.4 (n = 88)	0.16

EFS: event-free survival; OS: overall survival; RFS: relapse-free survival; u.d.: undefined; [a] 14 patients were excluded from analysis due to missing data on the percentage of CD33-positive leukemic blasts.

Supplemental Table S5. Effect of the percentage of CD33-positive leukemic blasts on survival (CC+CT vs TT).

CD33		OS			EFS			RFS		
		CC+CT	TT	Total	CC+CT	TT	Total	CC+CT	TT	Total
**0-10%**	*n*	6	2	8	6	2	8	6	2	8
	Median	78.6	u.d.	u.d.	9.6	61.5	17.6	8	60.8	16.2
**11-20%**	*n*	7	1	8	7	1	8	7	1	8
	Median	30.2	12.7	21.5	25.8	8.4	17.1	24.2	3.8	14
**21-30%**	*n*	2	0	2	2	0	2	1	0	1
	Median	79.4	-	79.4	64.4	-	64.4	u.d.	-	u.d.
**31-40%**	*n*	4	0	4	4	0	4	4	0	4
	Median	30.8	-	30.8	19.6	-	19.6	17.2	-	17.2
**41-50%**	*n*	3	3	6	3	3	6	2	2	4
	Median	15.6	4.7	13.3	7.9	2.6	5.3	61.5	7.8	61.5
**51-60%**	*n*	8	2	10	8	2	10	6	2	8
	Median	74.8	6.6	43.1	7.5	4.5	7	8.8	3.6	6.6
**61-70%**	*n*	2	2	4	2	2	4	2	2	4
	Median	5.8	38.7	5.8	5.8	38.4	5.8	5.1	36.8	5.1
**71-80%**	*n*	6	2	8	6	2	8	4	1	5
	Median	65.4	4.4	17.6	57.6	3.9	9.6	u.d.	7.2	u.d.
**81-90%**	*n*	19	2	21	19	2	21	15	1	16
	Median	19.2	4.4	13.5	9.7	2.9	8.6	10.8	4.8	9.9
**91-100%**	*n*	93	6	99	93	6	99	82	5	87
	Median	24.8	u.d.	27.1	8	54.2	8.2	9.3	u.d.	10.4
***p*-value**		0.49	0.99	0.53	0.52	0.86	0.57	0.7	0.52	0.86

EFS: event-free survival; *n*: number of patients; OS: overall survival; RFS: relapse-free survival; u.d.: undefined.

Supplemental Table S6.

A) Univariable model of OS, EFS and RFS (CC+CT vs TT).

	Univariable model								
	**Overall survival**			**Event-free survival**			**Relapse-free survival**		
**Characteristic**	HR	95% CI	*p*-value	HR	95% CI	*p*-value	HR	95% CI	*p*-value
CD33 SNP genotype									
CC+CT	-	-		-	-		-	-	
TT	1.16	0.64, 2.12	0.60	0.98	0.56, 1.70	>0.90	0.91	0.47, 1.75	0.80
% CD33-positive leukemic blasts	1.00	1.00, 1.01	0.70	1.00	1.00, 1.01	0.90	1.00	0.99, 1.01	>0.90
ELN risk (2022)									
Favorable	-	-		-	-		-	-	
Intermediate	2.11	1.25, 3.56	**0.005**	2.18	1.37, 3.48	**0.001**	2.28	1.39, 3.76	**0.001**
Adverse	2.85	1.71, 4.76	**<0.001**	2.56	1.61, 4.06	**<0.001**	2.20	1.32, 3.68	**0.003**
Sex									
Female	-	-		-	-		-	-	
Male	1.50	1.03, 2.19	**0.03**	1.69	1.19, 2.39	**0.003**	1.66	1.13, 2.46	**0.01**
Age at first diagnosis	1.03	1.01, 1.04	**0.003**	1.03	1.01, 1.04	**<0.001**	1.02	1.00, 1.04	**0.02**
CR after INDC1									
No	-	-		-	-		-	-	
Yes	0.40	0.27, 0.58	**<0.001**	0.36	0.25, 0.51	**<0.001**	0.67	0.42, 1.07	0.10

Multivariable model

B) Forest plot of hazard ratios from Cox regression analyses for OS, EFS, and RFS comparing CD33 SNP genotype TT versus CC+C.

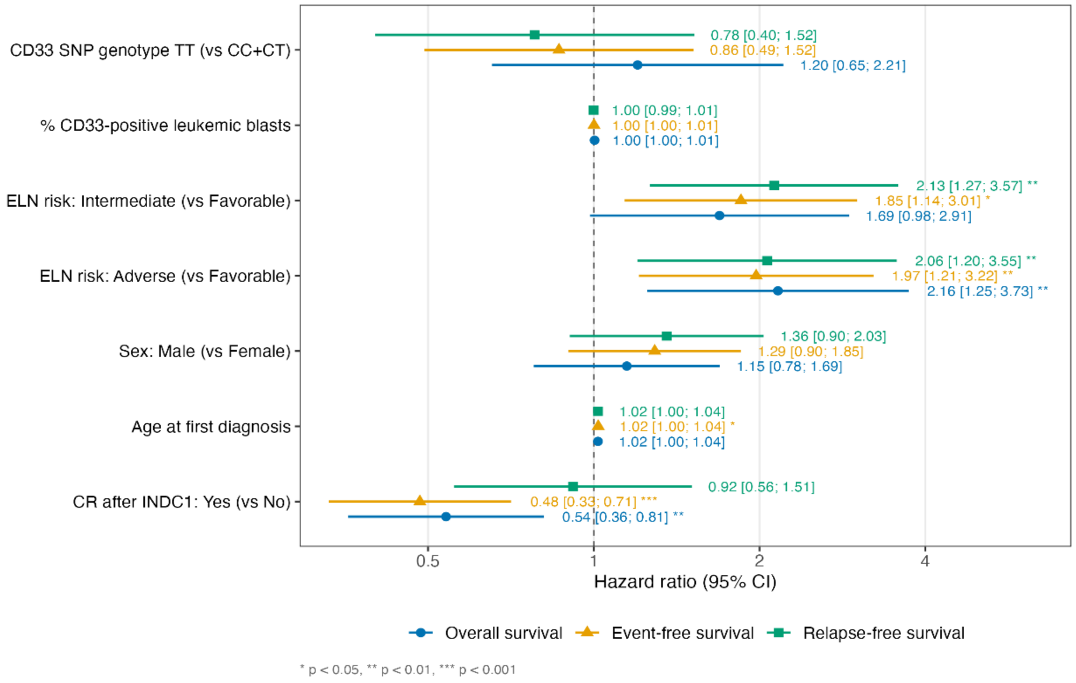

CI: confidence interval; CR: complete remission; EFS: event-free survival; ELN: European LeukemiaNet; HR: hazard ratio; INDC1: induction cycle 1; OS: overall survival; RFS: relapse-free survival; SNP: single nucleotide polymorphism.
